# Intermittent and Remittent Fevers

**Published:** 1852-09

**Authors:** 


*Intermittent and Remittent Fevers.*

We have included under the same head the above-named diseases because we cannot find, either in their symptoms or mode of treatment, sufficient reasons for regarding them as distinct diseases; in-indeed, so far as our observations extend, it is extremely difficult to determine whether a great majority of the so-called miasmatic diseases of this section of country present more of the symptoms as given in the books of an intermittent or a remittent fever.

The treatment which we have found most efficient in all cases, is the free use of quinine combined with opium or morphine, in proportions varying with the symptoms. When the intermissions are well marked, the quinine alone answers a good purpose. On the contrary, in proportion as the disease assumes the type of a remittent, and especially if the skin, kidneys, liver and other excretory organs are inactive, opium is required in combination, and in doses varying from 2 to 4 grs. with 5 to 8 grs. of quinine, 3 or 4 times daily.

Under this treatment the paroxysms are generally broken up in from 24 to 48 hours, after which the continued use of iron, with small doses of quinine and diluted nitro-muriat. acid, will, in most cases, restore the patient's health and strength in a week or ten days.

Chloride of sodium was used in several of the above cases to the exclusion of all other active remedies, and always with results which tend to confirm our previously expressed views concerning the remedial properties of this salt.

The use of active cathartics, and especially of mercurials, in this class of fevers, we believe to be not only useless, but absolutely injurious. In fact, it is our conviction, after fifteen years' experience in observing and treating the miasmatic diseases of the West and South, that the depleting and debilitating effects of active cathartics, as used by ignorant practitioners and in quack medicines, are as detrimental, if not more destructive to life and health, than the diseases for the cure of which they have been so indiscriminately administered.

In making these remarks we do not wish to be understood as condemning the use of cathartics. It is not to the proper use, but to the abuse of these, in many instances, most valuable remedial agents, that we object; for it must be admited by all that nothing can be more marked than the beneficial effects of a cathartic in dislodging often accumulated fecal and other irritatng matter from the intestines, or of a gentle laxative in stimulating to action the liver and small excretory glands of the alimentary canal.

In the treatment of the 20 cases of fever under consideration, a combination of rhubarb and carb. of soda was the only laxative used; its action, though not as rigorous, seemed to me even more efficient as a curative means than the strong cathartics, such as calomel and jalap, Cook's pills, &c., which, as an orthodox practitioner, we were bound to use during the time we were taking our initiatory steps in Western practice. The mode of action of this simple combination of rhubarb and soda is explained, to our satisfaction at least, in the article entitled "The Liver and its Diseases," published in the 1st number of the present vol. of this Journal. In confirmation of our views then expressed, we have Dr. Manley's experience, as given in the present number, article II. H.

